# Cell therapy for Parkinson’s disease with induced pluripotent stem cells

**DOI:** 10.1186/s41232-023-00269-3

**Published:** 2023-02-27

**Authors:** Asuka Morizane

**Affiliations:** 1 Department of Regenerative Medicine, Center for Clinical Research and Innovation, Kobe City Medical Center General Hospital, Kobe, Japan; 2 Department of Clinical Application, Center for iPS Cell Research and Application, Kyoto University, Kyoto, Japan

**Keywords:** Pluripotent stem cells, Parkinson’s disease, Transplantation, Cell therapy, Central nervous system

## Abstract

Parkinson’s disease (PD) is the second most common neurodegenerative disease and a prime target of cell therapies. In fact, aborted fetal tissue has been used as donor material for such therapies since the 1980s. These cell therapies, however, suffer from several problems, such as a short supply of donor materials, quality instability of the tissues, and ethical restrictions. The advancement of stem cell technologies has enabled the production of donor cells from pluripotent stem cells with unlimited scale, stable quality, and less ethical problems. Several research groups have established protocols to induce dopamine neural progenitors from pluripotent stem cells in a clinically compatible manner and confirmed efficacy and safety in non-clinical studies. Based on the results from these non-clinical studies, several clinical trials of pluripotent stem cell-based therapies for PD have begun. In the context of immune rejection, there are several modes of stem cell-based therapies: autologous transplantation, allogeneic transplantation without human leukocyte antigen-matching, and allogeneic transplantation with matching. In this mini-review, several practical points of stem cell-based therapies for PD are discussed.

## Introduction

Parkinson’s disease (PD) is the second most common neurodegenerative disorder and is especially common in the elderly. Medication with L-dopa is the standard treatment for PD, but its initial effectiveness gradually decreases with the progress of the disease. In these cases, increasing the L-dopa dose is one option, but the risk of side effects, such as on-off phenomena, wearing-off, and dyskinesia, increases. Deep brain stimulation (DBS) is an option for PD patients in the middle stage. Motor exercises are also a standard treatment. However, all these therapies for PD are symptomatic. Stem cell-based therapies for PD, the main interest of this review, on the other hand, have the potential to delay or reverse the progression of the disease.

## Overview of cell therapies for Parkinson’s disease

PD is histologically marked by the degeneration of midbrain dopamine neurons in the substantia nigra. Midbrain dopamine neurons extend neurites to the putamen and caudate, where they release dopamine as a neurotransmitter, but, because of the degeneration, the dopamine level of the putamen is decreased in PD. In cell therapies for PD, donor dopaminergic progenitors are normally injected into the putamen by ectopic transplantation, where they make local neural circuits with host neurons and become functional [1–3]. Transplanting dopaminergic neural progenitors to the substantia nigra by homotopic transplantation has also been tried [4, 5]. However, although some of the survived donor neurons in the substantia nigra extend neurites to the putamen, the functional benefit is inferior to ectopic transplantation.

Much of our knowledge about cell therapies for PD is based on previous clinical experiences of aborted fetal nigral transplants [1–3]. Many laboratories, including ours, transplant donor cells at the stage of immature progenitors [6–9]. These cells survive, mature, and extend neurites in the grafted region to make synaptic connections with the host neurons, a phenomenon that takes some time. Indeed, many in vivo studies showed that functional recovery starts several months after the transplantation. According to clinical reports on fetal nigral transplants, the recipient’s symptoms gradually recover over a period of more than 4 years and then plateau [10, 11].

## Induction of dopamine neurons from pluripotent stem cells

Many research groups have developed protocols to induce mesencephalic dopaminergic progenitors from pluripotent stem cells. Now, most protocols use small molecules to inhibit the BMP and TGF beta/Activin/Nodal pathways to specify neural fate, so-called dual SMAD inhibition [12]. To induce the floor plate, which is where mesencephalic dopamine progenitors originate, Sonic hedgehog (SHH) signaling is activated and Wnt signaling is adjusted normally with a GSK3 inhibitor [7, 8]. To purify the desired cell population, cell sorting technologies using specific antibodies, such as CORIN [13] and LRTM1 [14], have been adopted.

## Mode of transplantation

**Fig. 1:**
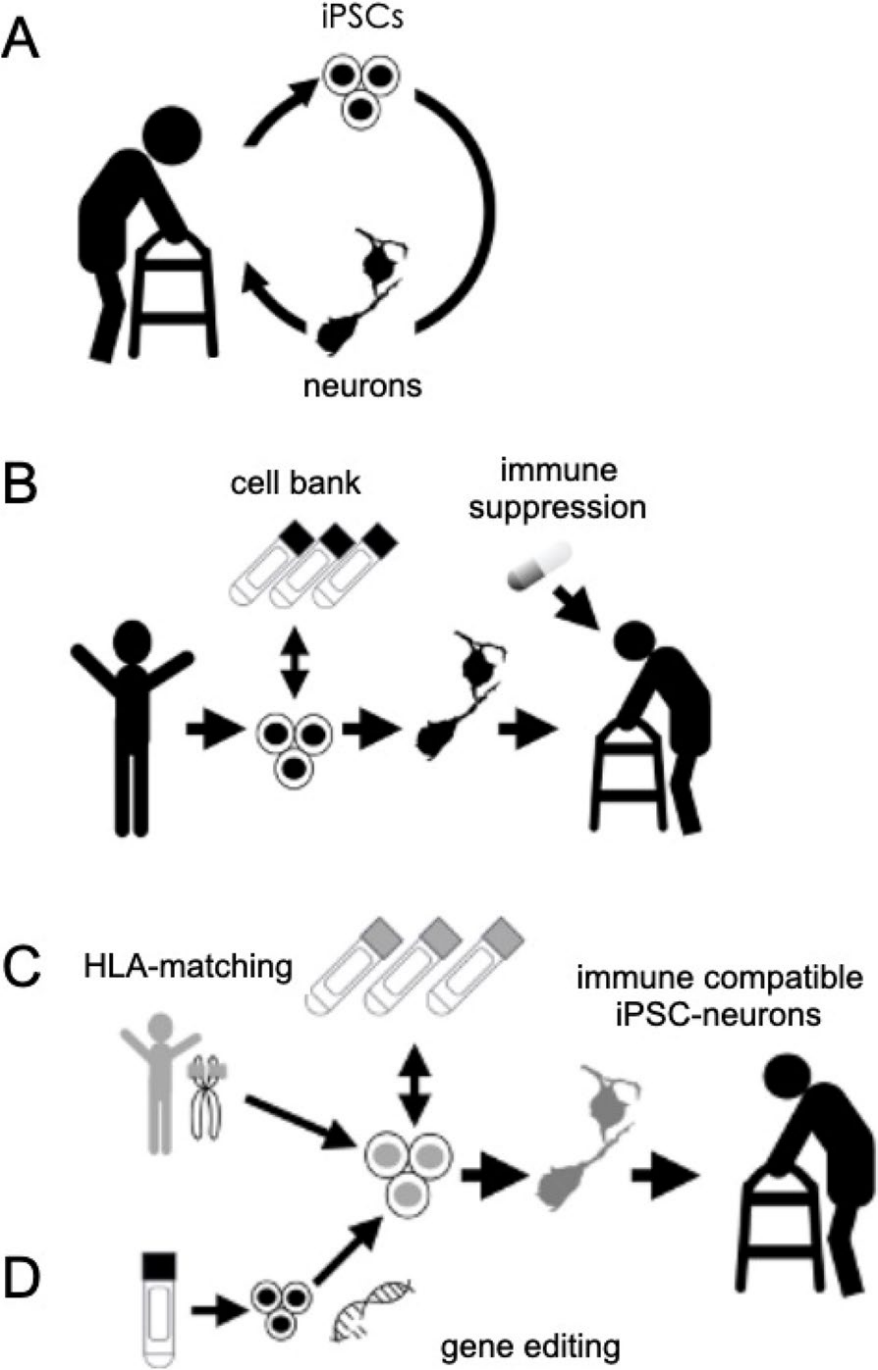
Modes of stem cell therapy. **A** Autologous transplantation with the patient’s own induced pluripotent stem cells (iPSCs). **B** Allogeneic transplantation with iPSCs from healthy donors or human embryonic stem cells without HLA-haplotype matching. **C** Allogeneic transplantation with HLA-matching iPSCs. **D** Allogeneic transplantation with “universal cells” that are genetically edited to knock down HLA molecules

The risk of immune rejection should be considered generally in cell therapies. Although the brain has been considered a site of immune privilege, this concept is being revisited [15]. Past clinical experience of fetal cell transplantation has shown that the use of immune suppressants improves the outcome [3]. Stem cell-based transplantations provide several modes in the context of immune rejection: autologous transplantation (Fig. 1A), allogeneic transplantation without human leukocyte antigen-matching (Fig. 1B), allogeneic transplantation with matching (Fig. 1C), and finally the application of “universal” pluripotent stem cells (Fig. 1D). Universal pluripotent stem cells describe pluripotent stem cells gene-edited so that their expression of HLA molecules has been suppressed or knocked out. This feature allows them to escape from the major HLA-related immune response of the host, but immune responses related to NK cells and minor antigens need to be also considered. To minimize the NK cell response, several strategies have been reported [16–19]. Ultimately, these universal donor cells are expected to reach clinical application, because, theoretically, they are best suited to avoid a graft-related immune response. In the meantime, the only clinical trials of pluripotent stem cell-based therapies for PD have used allogeneic transplantation with immune suppressants [9, 20] or autologous transplantation [21].

## Clinical practice of cell therapies for Parkinson’s disease

### Surgical indications

Because medication is effective at controlling symptoms in the early stages of PD, new interventions are not recommended in these cases. After confirming the safety and efficacy of a new intervention by clinical trials with focused patients, the indication for surgery should be expanded step by step. Reports and experiences from previous clinical studies of aborted fetal transplantations can help determine which patients will benefit from cell therapy. Accordingly, cell therapy is not promising for extra-advanced PD. When the patient is still responsive to L-dopa, i.e., postsynaptic neurons are preserved and can respond to the released dopamine, cell therapy is considered a good indication. For an ongoing investigator-initiated clinical trial in Japan [20], the criteria include patients aged 50–70 years old, disease duration of more than 5 years, Hoehn and Yahr stage ≧ III at off period and ≦ III at on period, and a levodopa response more than 30%. The general condition of the patients should be taken into consideration because the patients need to withstand the surgical operation and immune suppression afterwards. Particularly, the susceptibility to infection or renal dysfunction should be carefully considered, as these conditions may limit the use of immune suppressants.

### Stereotactic surgery

The donor cells are injected directly into the brain as cell suspensions or cell clumps through a transplantation needle. In clinical trials in Japan, the cells were cultured for two weeks after cell sorting to form clumps, which were transplanted without dissociation [7]. For PD, a similar stereotactic brain surgery system used for DBS can be applied for the cell injection at the target site.

### Number of cells to transplant

Previous reports of autopsy brains with aborted fetal transplants have shown that the survival of 43,000–138,000 dopaminergic neurons in the unilateral striatum is needed to improve the symptoms. Since there are 250,000 dopaminergic neurons projecting to one putamen in the human brain, the goal of cell therapy is to innervate about half that number, or 100,000 of the grafted dopaminergic neurons. Acknowledging this number of cells must survive, the number of cells needed for the transplantation is reverse engineered and depends on the purity of the donor populations, the survival rate, and the environment of the host brain. Our group transplanted human iPSC-derived dopaminergic progenitors into monkey brains and examined the brains histologically 2 years later. About 2.4 million cells were transplanted unilaterally, and an average of 64,000±49,000 dopaminergic neurons survived [6]. Dopaminergic neurons in the substantia nigra of the midbrain, which are affected in PD, are a very small fraction of the total number of neurons in the brain. Because the number of cells for this therapy is much smaller than the number required for cell therapies of the heart, muscle, or liver, PD is a suitable initial target for cell therapy-based regenerative medicine.

### Quality control of donor cells

To ensure optimal efficacy and avoid risks in cell therapy, the quality of donor cells needs to be controlled with appropriate strategies. In the case of the pluripotent stem cell-based approach, quality control (QC) is performed at each stage: undifferentiated cells, cells in the process of differentiation, and final cell products. It is necessary to check morphology, pluripotent markers, infectivity, and toxicity. In addition, it might be expected to analyze genetic and epigenetic mutations to assess the risk of tumorigenesis. The risk of tumorigenesis can be categorized as follows: (i) malignant tumorigenesis caused by gene or chromosome mutations, so-called oncogenesis; (ii) teratoma formation due to residual undifferentiated cells, or the possibility of forming another tissue component derived from undesired cells; (iii) neural overgrowth caused by too immature neural cells. In the QC for the final cell product in the Japanese clinical trial, the markers of dopamine progenitors (FOXA2/TUJ1), undifferentiated cells (POU5F1, LIN28, OCT3/4/TRA2-49/6E), and early neural markers (SOX1/PAX6) were analyzed [20]. In the pre-clinical study of the Japanese trial, the donor cells, which had passed the QC tests, were transplanted into animals and their safety was confirmed after long-term observation [6, 7, 20].

### Immune suppression

Previous clinical trials of aborted fetus transplantations for PD included a mixture of tissues from several abortions with a variety of HLA haplotypes. In those trials, immune suppression with a triple regimen, i.e., cyclosporine, prednisolone, and azathioprine, was normally used. Even then, immune tolerance was established only 1 to 2 years after the transplantation, and the immune suppressive drug treatment was stopped 2 to 3 years after the transplantation [11]. The iPSC-based trial in Japan described above uses only a single cell line which expresses the most frequent HLA haplotype in Japan; thus, it is less likely to cause an immune reaction than past fetal transplants. Therefore, immune suppression with a single drug, tacrolimus, is being used in the trial.

In addition, a PET scan using a translocator protein (TSPO) tracer is used to monitor the graft-derived inflammatory response, because TSPO is a tracer that accumulates in activated microglia and can capture inflammation in the brain. Indeed, TSPO PET can detect graft-derived inflammatory responses in the monkey brain [22, 23]. However, tacrolimus has side effects, such as increased incidences of cancer and diabetes, renal dysfunction, and susceptibility to infection; thus, it requires frequent monitoring of its blood concentration.

## Future perspective

**Fig. 2:**
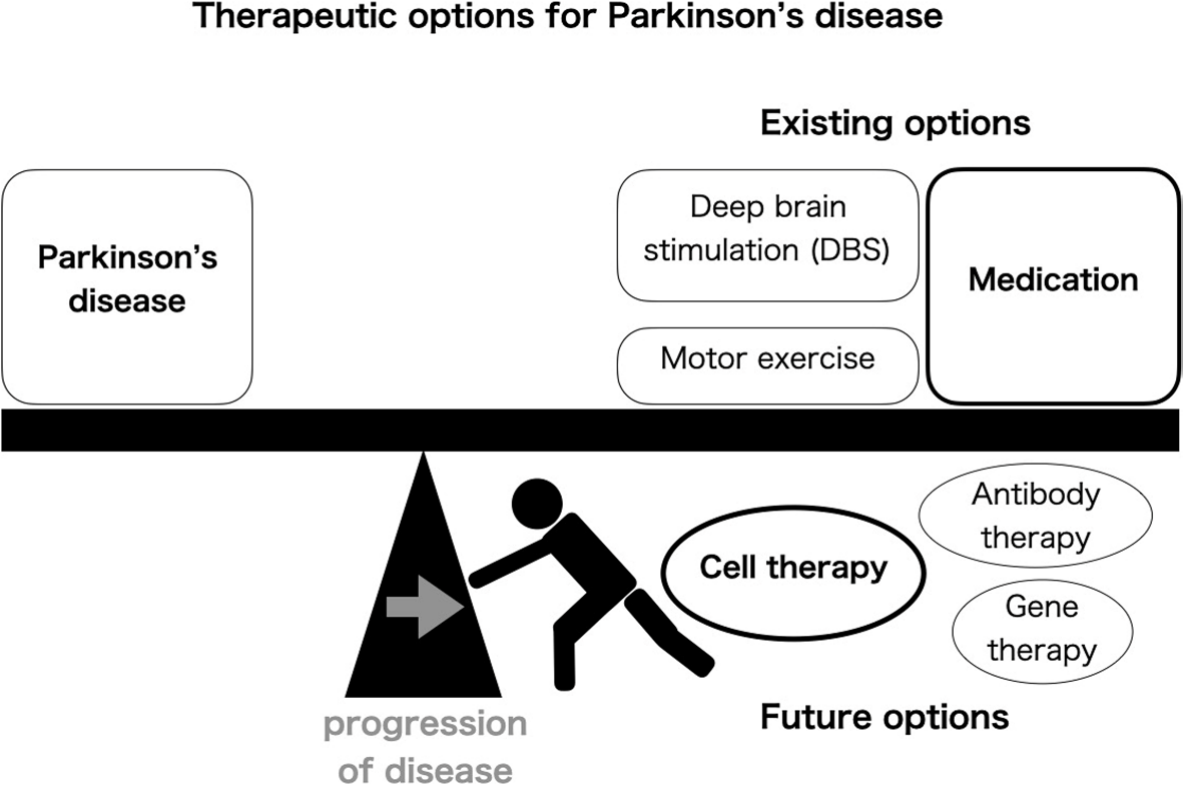
Therapeutic options for Parkinson’s disease. PD is a progressive disease. Existing therapeutic options include medication, DBS, and motor exercise, but all are symptomatic therapies. Cell therapies for PD are expected to become a standard therapeutic option in the future that can stop or reverse the disease progression

Further development and research are necessary to expand cell therapies as a standard treatment for PD. To supply a large amount of “cell product” in a stable manner, it is necessary to automate cell processing in a completely closed system. Universal cells that are less prone to immune reactions are being developed as cell therapies not only for PD but also for other diseases and other organs. With more technological breakthroughs, autologous cells could be prepared efficiently at a lower cost. Thus, in the future, cell therapies are expected to join medication, DBS, and motor exercise as standard PD treatment (Fig. 2).

## Conclusion

Clinical application of pluripotent stem cell-based therapies for PD has started. After showing safety by the initial clinical trials and following several improvements, cell therapies for PD would become a standard clinical option in the future.

## Data Availability

Not applicable.

## Abbreviations

iPSC: Induced pluripotent stem cell
PD: Parkinson’s disease
DBS: Deep brain stimulation
SHH: Sonic hedgehog
TSPO: Translocator protein

## Declarations

### Competing for interests

The author receives a grant for collaborative research from Sumitomo Pharma Co., Ltd.
